# Acquired unilateral alopecia after arterial infusion chemotherapy in a recurrent nasopharyngeal carcinoma

**DOI:** 10.1002/cnr2.1671

**Published:** 2022-07-21

**Authors:** Bao‐Feng Duan, Hua‐Ying Chen, Xue‐Mei Zheng, Qing He

**Affiliations:** ^1^ Department of Head and Neck Oncology West China Hospital of Sichuan University Chengdu Sichuan Province China

**Keywords:** adverse effects, alopecia, interventional radiology, nasopharyngeal carcinoma, radiation therapy

## Abstract

**Background:**

Intractable nasopharyngeal hemorrhage is a severe complication with high mortality rate in patients with radiation therapy (RT) for nasopharyngeal carcinoma (NPC) that requires emergency treatment. Quite a few of them combine with tumor recurrence. Treatment planning for these patients is extremely difficult for oncologists, and effective treatments are lacking.

**Case:**

A 42‐year‐old man had a history of recurrent NPC that was treated with 2 cycles of chemoradiotherapies from 2017 to 2019. Five months after the second round of chemoradiotherapy, an episode of massive nasal bleeding occurred. As positron emission tomography (PET) scan revealed tumor recurrence in the left wall of nasopharynx, superselective embolization and subsequent intra‐arterial infusion (IA, 4 times of cisplatin 60 mg + fluorouracil 1.0 g) were performed to stop bleeding and achieve tumor control. To date, the disease‐free survival time has been over 1 year. No tumor recurrence or rebleeding is found except for alopecia on the left side.

**Conclusions:**

Interventional radiology is important and effective in the treatment of recurrent NPC for both massive nasal bleeding and tumor control. However, the unique complication of unilateral alopecia should not be ignored.

## 1 INTRODUCTION

Irradiation and reirradiation are the mainstay of curative therapies for NPC. However, they may lead to substantial severe complications, including fatal hemorrhage. In addition to radiation injury, tumor recurrence is believed to be a significant cause of this critical condition.^1^, ^2^, ^3^ Treatments for these patients are lacking. Interventional radiology has been widely used in the treatment of massive nasal bleeding after chemoradiotherapy for NPC, and this technique is also an appropriate choice to safely achieve tumor control. However, it is seldom recommended. In this case, interventional radiology was used not only to stop bleeding but also to achieve tumor control. Finally, a satisfactory result is obtained.

### 1.1 Case

In 2017, this 42‐year‐old man presented to our hospital with a 6‐month history of blood‐tinged sputum. Nasopharyngeal biopsy revealed low‐differentiated nonkeratinized squamous cell carcinoma in the nasopharyngeal area. Then, he was diagnosed with NPC (T2N1M1, stage IV C). The initial chemoradiotherapy cycle was four courses of Docetaxel (75 mg/m^2^) 100 mg d1；cisplatin (75 mg/m^2^) 40 mg d1, 30 mg d2‐3；fluorouracil (2400 mg/m^2^) 600 mg d1‐5 q3w followed by Concurrent radio‐chemotherapy of cisplatin (100 mg/m^2^) 50 mg d1，40 mg d2‐3, and volumetric intensity modulated arc therapy 69.96 Gy, 33 fractions. After that, the evaluation of tumor response was complete response. In 2019, blood‐tinged sputum recurred, and nonkeratinized squamous cell carcinoma was detected again in the left nasopharyngeal area. Therefore, local recurrence of NPC was diagnosed. The second cycle of chemotherapy was one course of gemcitabine (1000 mg/m^2^) 1.2 g d1, 8 and cisplatin (75 mg/m^2^) 40 mg d1, 30 mg d2‐3 followed by concurrent radio‐chemotherapy of cisplatin (100 mg/m^2^) 50 mg d1，40 mg d2‐3 and intensity modulated radiation therapy, imaging‐guided radiation therapy 66 Gy, 33 fractions. However, the patient refused to continue chemotherapy because of intolerable headache and hearing defect.

**FIGURE 1:**
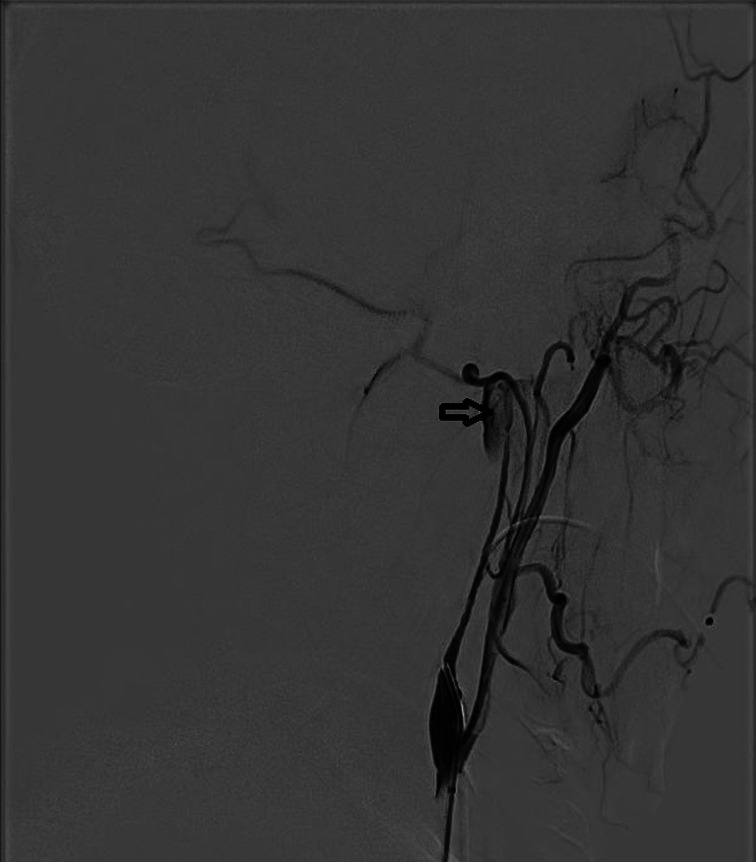
Angiography showed a pseudoaneurysm at the petrous portion (C2 segment) of the left ICA (arrow)

**FIGURE 2:**
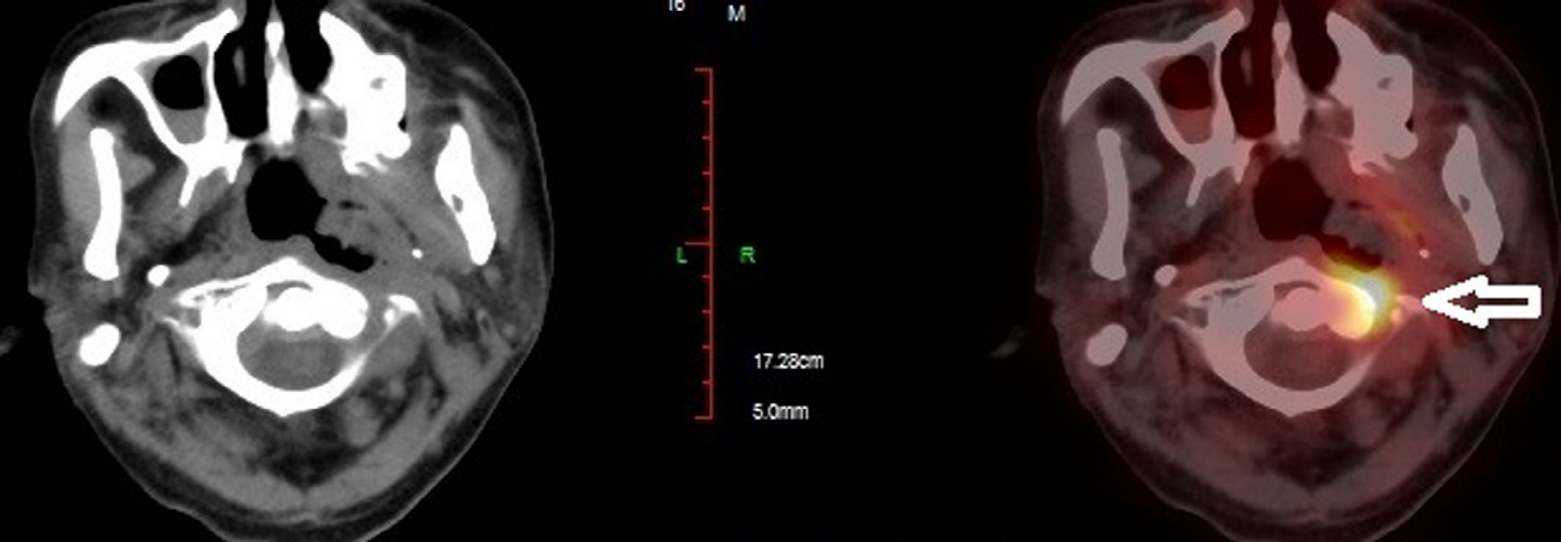
Patient's imaging results before IA. The arrow indicates the tumor area. PET showed a soft‐tissue lump in the left wall of the nasopharynx (maximum standardized uptake value 6.51)(arrow). The boundary of the lesion was unclear. The mass invaded and destroyed the adjacent bone

**FIGURE 3:**
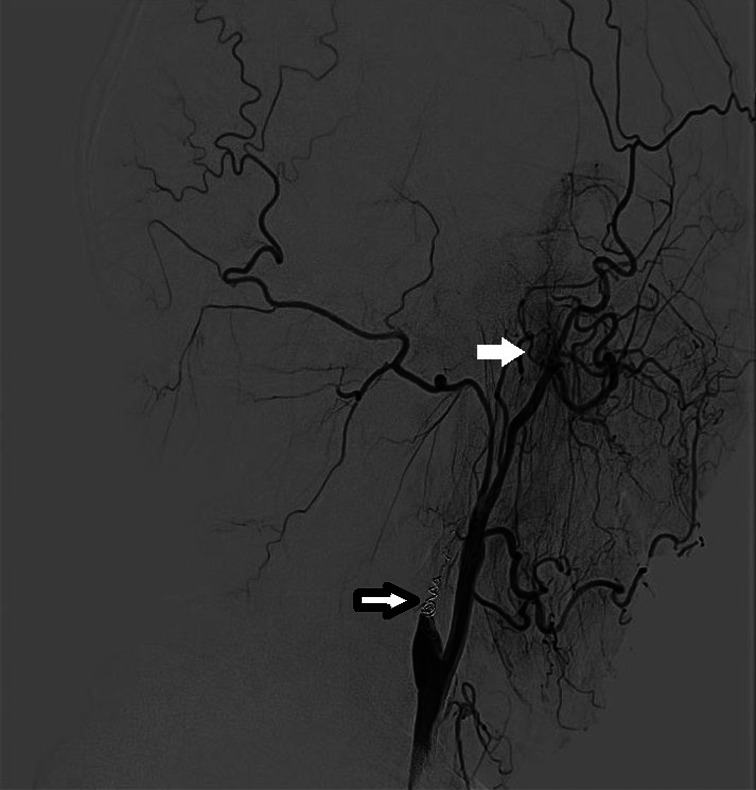
Lateral view angiogram of the first IA showed tumor vascularity (white arrow) and total occlusion of the ICA after coil embolization (black arrow)

**FIGURE 4:**
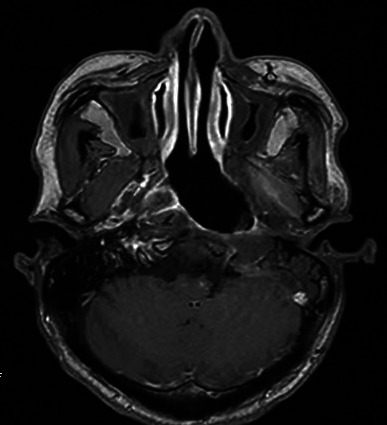
Patient's imaging results 8 months after the fourth IA. Enhanced MRI revealed that there were no signs of tumors

**FIGURE 5:**
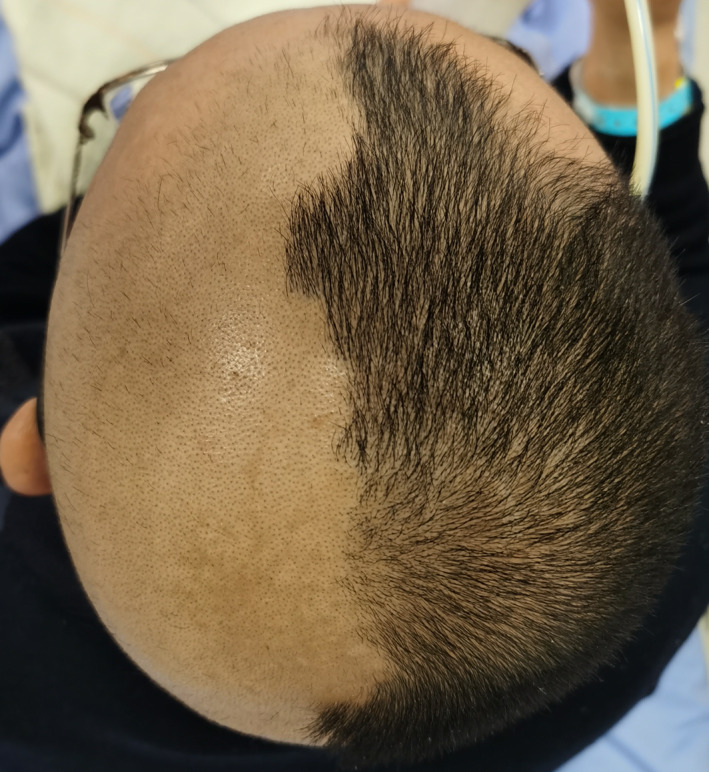
Unilateral alopecia after IA.

Five months after the second round of chemoradiotherapy, an episode of massive nasal bleeding occurred after the patient contracted a cold. Despite performing anterior and posterior nasal packing, approximately 700 ml of blood was discharged from his mouth and nasal cavity within 24 h. Emergent angiography was performed. A ruptured pseudoaneurysm was identified in the petrous portion (C2 segment) of the left internal carotid artery (ICA) where the artery was completely interrupted (Figure 1). Fortunately, complete cessation of bleeding was successfully achieved with coil embolization. Subsequently, a PET scan revealed local recurrence of the tumor in the left nasopharyngeal area (Figure 2). According to the consultation of our NPC multidisciplinary team, the patient was not amenable to palliative radiotherapy and surgery. Therefore, we arranged the IA as it was still life‐threatening with tumor recurrence if the patient was treated only with best supportive care according to the NCCN. Then, chemical infusion into the left external carotid artery (ECA) was performed four times with cisplatin 60 mg plus fluorouracil 1.0 g once a month to achieve tumor control (Figure 3). A total of 12 months later, there was no rebleeding and MRI showed that there were no signs of tumor recurrence (Figure 4). Interestingly, the patient complained of alopecia on the left side 1 month after the first IA (Figure 5), which lasted for 12 months before his hair came to recover. As the alopecia was reversible and occurred on the same side, the IA procedure was performed on, we made a diagnosis of IA‐related alopecia.

## 2 DISCUSSION

Currently, radiation is widely used as the first‐line treatment for NPC and greatly improves patient survival. Nevertheless, radiation also places patients at risk of radiation‐induced adverse effects, the most fatal of which is massive nasal bleeding.^4^, ^5^ These recurrent NPCs with massive nasal bleeding are usually dangerous and critical. Their treatment is extremely difficult, and effective treatments are lacking.^6^, ^7^, ^8^ Previous reports on these patients mainly focused on hemostasis strategies, few took notice of further tumor control, which was also crucial to these patients.^9^, ^10^

Interventional radiology has been demonstrated to be safe and effective in the treatment of massive nasal bleeding after radiotherapy for NPC. Furthermore, superselective IA chemotherapy is believed to be superior to intravenous injection because higher concentration and lower dose of chemotherapeutic agents can be delivered directly to the tumor site. By this means, systemic toxic reactions can be minimized, while antitumor effect can be maximized. Thus, interventional radiology is recommended by the NCCN for many solid tumors, such as hepatocellular carcinoma and colorectal cancer and melanoma. Since NPC tends to localize and metastasize to regional lymph nodes without general metastases, this technique should be an appropriate choice to safely achieve better tumor control.^11^ However, it was seldom suggested to these patients.

The performance status (PS) score of this patient was 3 because of extensive blood loss and severe infection. According to the NCCN, locoregional recurrent NPC with prior RT on the condition of PS score 3 was recommended to receive best supportive care plus palliative RT or palliative surgery. However, the consultation of oncologist and surgeon indicated that he was no longer eligible for RT and surgery. Therefore, we arranged IA right after the embolism of ICA, as the existing tumor was still life‐threatening if the patient was treated only with best supportive care. To date, no signs of rebleeding or tumor recurrence have been found.

The main hemostasis strategies of interventional radiology were embolism and stenting. The major side effects are cerebral ischemia (approximately 10%–25%) depending on collateral blood flow.^12^ Compared to ICA embolism, the side effects of IA in ECA have seldom been reported. In the present case, the only significant complication of IA was unilateral alopecia, and this unique alopecia was reversible. In fact, there have been no previous reports of alopecia correlated with IA in ECAs found in PubMed.

It is well known that chemotherapy causes alopecia however, it is less well known that hair follicles are more strongly affected when the chemicals are infused directly into the external carotid artery. Furthermore, alopecia caused by chemical infusion into the external carotid chemical artery is unilateral. Therefore, warning patients of the risk of unilateral alopecia is necessary when IA into the external carotid artery is planned.

In summary, the technique of transcatheter IA in the ECA should be a promising method with acceptable side effects for relapsed NPCs with massive nasal bleeding and is worth further prospective studies to improve the quality of evidence.

## AUTHOR CONTRIBUTIONS

**Bao‐Feng Duan:** Conceptualization (equal); data curation (equal). **Hua‐Ying Chen:** Data curation (supporting). **Xue‐Mei Zheng:** Data curation (supporting). **Qing He:** Conceptualization (equal); writing – original draft (equal); writing – review and editing (equal).

## CONFLICT OF INTEREST

The authors declare that they have no conflicts of interest.

## Data Availability

Data will be made available upon reasonable request
